# A Case of Gaucher’s Disease in a Boy Thirteen Years of Age

**Published:** 1914-07

**Authors:** 


**A Case of Gaucher's Disease in a Boy Thirteen Years of Age. Splenectomy with Recovery.** Herman, Roth and Bernstein bring in Archives of Pediatrics, May, 1914, a case of Gaucher's disease which recovered after operation, this being the ninth operative case and the sixth recovery after splenectomy.

C. G. L-W.

